# Predictors of swimming pool supervision for caregivers of toddlers

**DOI:** 10.5249/jivr.vo113i2.1661

**Published:** 2021-07

**Authors:** Molly B Johnson, Elizabeth D Boriack, Carlee M McConnell, Stewart R Williams, Jessica A Naiditch, Karla A Lawson

**Affiliations:** ^*a*^ Trauma and Injury Research Center, Dell Children’s Medical Center, Austin, TX, USA.; ^*b*^ Kinesiology Department, University of the Incarnate Word, San Antonio, TX, USA.; ^*c*^ Trauma Services, Dell Children’s Medical Center, Austin, TX, USA.; ^*d*^ Department of Surgery and Perioperative Care, University of Texas at Austin, Austin, TX, USA.

**Keywords:** Drowning, Submersion, Water safety, Behavior, Attitudes

## Abstract

**Background:**

In the U.S., drowning is a leading cause of death for toddlers. One important layer of protection against submersion injuries and fatalities is parent or caregiver supervision. The aims of this study are to explore current supervisory behavior of caregivers, determine how caregivers view com-mon supervision distractions, like cell phones and grilling, and identify what factors shape the quality of supervision that is given when swimming with their toddler at a swimming pool.

**Methods:**

This cross-sectional study used the MTurk online platform to survey 650 caregivers of toddlers (1-4 yrs old) about their supervision behavior, their drowning knowledge, their perceptions of arm’s reach supervision, the water competency of their toddler, and other background information. Regression analysis was used to identify factors that predicted report-ed supervision behavior.

**Results:**

The average supervision behavior score for caregivers indicated an attitude between neutral and disagreement with allowing distractions for themselves while supervising their toddler in a swimming pool. High water safety knowledge and positive perceptions of arm’s reach supervision were the biggest predictors of attentive supervision behavior. Having a home pool, higher education level, and believing their toddler had greater water competency were predictive of less attentive supervision behavior.

**Conclusions:**

Results suggest that supervision behavior while toddlers are in a swimming pool may be inade-quate. Low water safety knowledge and attitudes about what constitutes quality supervision are related to pool supervision behavior and changing these may reduce drowning risk. Caregivers should be encouraged to not reduce supervision as their toddlers gain water competency and if they have a home pool.

## Introduction

In the United States (U.S.), unintentional injuries are the leading cause of death for children. Drowning is one of the most common causes of unintentional death. In 2018, 14% of pediatric injury fatalities were due to drowning. For children 1-5 yrs old, drowning was the leading cause of unintentional death and accounted for 33.5% of all fatalities due to unintentional injuries.^1^ Globally, toddlers are at the highest risk of drowning compared to other age groups.^2^ Drowning is not always fatal, however. For every one pediatric drowning fatality in the U.S., five children are treated for non-fatal submersion injury. Additionally, drowning causes a high rate of morbidity, with 50% of patients needing admission to the hospital compared with 6% in other unintentional injury categories.^3^

In the U.S., most drowning incidents for toddlers occur in swimming pools, with home pools posing a particularly high risk. In 2014, 54% of infants and toddlers who drowned were in swimming pools, 19% were in natural water, 12% were in bathtubs, and 15% were in an unknown location. Of the pool drownings, 87% occurred in private pools, of which 59% occurred at the child’s own home.^4^ Further research is needed to understand factors that impact drowning risk where the majority of toddler drownings occur: in swimming pools. Additionally, we need a better understanding of factors that contribute to the high risk of drownings in home pools so we can develop effective countermeasures.

The American Academy of Pediatrics (AAP) recommends using layers of protection for drowning prevention that include wearing life jackets; installing 4-sided isolation fencing around home pools; swimming where lifeguards are stationed; developing water competency and swim skills; and providing close, constant, and attentive supervision.^5^ The time frame for instituting layers of protection varies; many decisions, like building fencing or taking cardiopulmonary resuscitation (CPR) classes, occur separate from swim time. Supervision is unique because it occurs during swim time and involves second-by-second decision-making in order to be constant. The possibility of planned activities (e.g. needing to prepare food) or unexpected distractions (e.g. receiving a phone call) interfering with supervision may change day-to-day and may vary greatly between caregivers. Research is needed to understand how supervision decisions may be impacted by common activities that can withdraw attention from supervision.

Despite the importance of supervision, it is common for parents of toddlers to not maintain constant supervision of children around bodies of water.^6^ This is a hazardous practice since a lack of supervision is one of the main risk factors associated with drowning.^7^ A study of fatal child drownings in Australia showed that a lack of supervision was a contributing factor in 94% of the cases where the supervision information for the incident was known.^8^ A study of fatal child drownings in the U.S. indicates that poor quality supervision was involved in most incidents.^9^ To improve drowning prevention messaging and programming for parents and caregivers, we need to develop a better understanding of what factors contribute to inadequate supervision when caregivers are responsible for their toddler in a swimming pool.

It is possible that many caregivers lack knowledge about how drowning occurs and how to prevent it. Research suggests that knowledge of drowning risk and prevention among parents and caregivers is poor.^6,10^ A survey of parents in the U.S. showed that 48% thought they would hear a child if they were drowning when drowning is actually often quick and silent.^4,5^

The AAP suggestion that swimming supervision be close, constant, and attentive is often referred to as involving “touch supervision” or arm’s reach supervision for young children.^5^ Despite the common recommendation to maintain arm’s reach supervision, not a lot is known about parents’ perceptions of this type of supervision and how that impacts actual supervision behavior.

Previous research surveying parents as their children progressed through swimming lessons shows that they may modify their supervision based on how much water competency or swimming skill they believe their toddler has.^11,12^ There is little research assessing whether a caregiver’s belief about their toddler’s water competency is predictive of supervision behavior when assessed unrelated to a course of swimming lessons.

In addition, research is beginning to look at what factors shape swimming supervision behavior, with findings from observational studies showing that caregiver age, child age, and the number of children in a group are key predictors of supervision behavior at the beach and in public pools, and that swimming ability is also predictive of supervision at public pools.^4,13^ It is not well understood how home pools impact supervision.

The aim of this study is to understand what factors are most predictive of how attentive caregivers will report being when supervising their toddler at a swimming pool. We hypothesized that high water safety knowledge, positive perceptions of arms reach supervision, and caring for toddlers believed to have few water competency skills or who have not had swimming lessons would be predictive of more attentive supervision behavior. We hypothesized that less attentive supervision would be reported by older caregivers and for older toddlers. 

## Methods 

**Survey procedures**

A cross-sectional study using an anonymous survey of caregivers of toddlers was conducted in August, 2020. Participants were recruited using the online Amazon MTurk platform, which is a common academic resource that offers access to a large participant pool, has been found to be representative of the general U.S. population, and is a source of quality research data.^14^ Only Amazon MTurk workers who lived in the U.S. and had a 95% or higher approval rating on prior projects could view the recruitment information. MTurk workers were only allowed to begin the survey if they answered that they were over 18 years of age and were the caregiver for a toddler (1-4 yrs old). Participating MTurk workers were paid $2 for completing the survey. The study was supported through internal hospital funding. The study was approved by the University of Texas at Austin Health Sciences Institutional Review Board. Participants reviewed and agreed to an informed consent document before starting the survey. 

The survey asked about the demographics and background of the caregiver and toddler, assessed drowning knowledge, and rated agreement with statements about perceptions of arm’s reach supervision and agreement with statements about supervision behavior related to attention and distraction when swimming with their toddler. 

Sample size calculations estimated a need for between 380 and 692 participants in order to detect a 10% difference in mean perceptions of arm’s reach supervision scores between those individuals with low and high behavior scores, with a 90% power and alpha of 0.05. Sample number estimate variations were due to estimated differences in score variation (standard deviation estimates of low variation and high variation). Surveys were completed for 916 participants. To ensure high quality data from the MTurk survey takers, a score was created that summed a number of poor data quality indicators, such as a completion time of less than 5 min, nonsensical textbox answers, and contradictory answers on multiple choice questions. Before analysis, 266 surveys with a poor quality indicator score were removed from the dataset. Data were analyzed for the remaining 650 participants. 

**Swimming supervision behavior scoring**

Caregivers were asked to rate their agreement with statements about distractions and attention while supervising their own toddler in the pool using a 5-point likert scale from strongly disagree to strongly agree. Half of the questions were worded positively so that agreement indicated attentive supervision. The other half were worded negatively so that agreement indicated distractible supervision. Agreement scores for negative statements were reversed and the ten statement scores were summed to create a single supervision behavior score that had a possible range of 10 - 50 where 10 reflects distractibility and 50 reflects attentiveness reported by caregivers while supervising their toddler in a swimming pool. 

**Perceptions of arm’s reach supervision scoring**

Caregivers were asked to rate their perceptions of arm’s reach supervision for toddlers using a 5-point likert scale from strongly disagree to strongly agree on ten statements that included various scenarios (e.g. Toddlers should be kept within arm's reach of their parent or caregiver even if they are in water that is not above the toddler's head). All statements were worded in a parallel manner so that agreement reflected that caregivers should keep toddlers within arm’s reach in each scenario. The ten statement scores were summed to create a single perceptions of arm’s reach supervision score that had a possible range of 10 - 50 where 10 reflects strong disagreement with arm’s reach supervision in all given scenarios and 50 reflects strong agreement with arm’s reach supervision in all given scenarios. 

**Drowning and water safety knowledge scoring**

Caregivers were given ten statements designed to test their knowledge related to drowning and water safety. They could answer True, False, or I don’t know for each statement. A total knowledge score was created by summing all correct answers. There was a possible range of scores from 0 to 10.

**Toddler water competency scoring**

Caregivers were asked whether their toddler was able to perform six water competency skills recommended by the American Red Cross: Enter water above his/her head and return to the surface; Float unassisted for 1 minute; Tread water for 1 minute; Turn around and then find an exit from the pool; Swim 25 meters without stopping (80 feet, the length of a standard pool); and Exit the pool without using a ladder.^15^ They could answer Yes, No, or I don’t know for each skill. A total water competency score was created by summing all Yes answers. There was a possible range of scores from 0 to 6.

**Survey development and validation**

The survey tool was developed based on a literature review of the topic. The survey was tested on caregivers of toddlers during development. Face validity was assessed through reviews by three experts in the field. Before analyzing the results, item analyses were performed on the supervision behavior section and the perceptions of arm’s reach supervision section to assess the reliability of each statement in relation to the other statements. For both the supervision behavior and perceptions of arm’s reach supervision sections, Pearson correlations showed moderate correlations for most pairs of statements. No one statement received low correlations with all other statements, indicating that no statement was inconsistent with the rest.

To assess the validity of the supervision behavior section score, an ANOVA determined differences in mean supervision behavior scores between Yes and No answers to, Do you think it is possible to keep your attention on your toddler when they are in the pool? A significantly lower mean supervision behavior score for those who answered No (31.36, SD: 4.58) compared to those who answered Yes (36.20, SD: 7.70) supported the validity of the supervision behavior section (F=5.49, p=0.0194). To assess the validity of the perceptions of arm’s reach supervision section score, an ANOVA determined differences in perceptions of arm’s reach supervision scores between Yes and No answers to, Do you think it is necessary to keep toddlers within arm’s reach when they are in the pool regardless of the situation? A significantly lower perceptions of arm’s reach supervision score for those who answered No (34.70, SD: 7.63) compared to those who answered Yes (39.68, SD: 6.82) supported the validity of the perceptions of arm’s reach supervision section score (F=22.44, p<0.01). 

**Data analysis**

The perceptions of arm’s reach supervision score, the knowledge score, the toddler water competency score, and 11 demographic or background survey questions were analyzed as possible predictors of supervision behavior. Supervision behavior scores for the participants were checked for normal distribution. Pearson correlation analyses were performed between all continuous variables to check for collinearity using a cutoff of r^2^=0.7. Variables with significant differences in supervision behavior score at p<0.05 on univariate analyses (Table 1) were included in a multivariate linear regression model with the supervision behavior score as the dependent variable. Scoring of knowledge, perceptions of arm’s reach supervision, water competency, and supervision behavior sections was performed in Excel. All statistical analyses were conducted using STATA SE version 12.0. 

**Table 1:** Participant background and mean supervision behavior score.

Variable	Category	n	%	Mean	SD
Gender*	Female	248	38.2	37.32	8.11
	Male	402	62.1	35.34	7.31
Age*	18-24 yrs	23	3.5	34.04	6.18
	25-34 yrs	352	54.2	35.61	7.44
	35-44 yrs	200	30.8	37.94	8.08
	45-54 yrs	47	7.2	34.49	7.26
	55 yrs or older	28	4.3	33.46	7.08
Race/ethnicity*	White	461	70.9	36.27	7.87
	Black	118	18.2	33.52	6.24
	Hispanic/latino	31	4.8	38.61	6.76
	Asian	18	2.8	41.39	7.58
	Mixed Race	22	3.4	38.36	7.75
Household income*	Less than $25,000	44	6.8	36.30	7.28
	$25,000-$49,999	197	30.3	35.60	7.19
	$50,000-$99,999	330	50.8	35.65	7.86
	$100,000 or more	79	12.2	39.11	7.74
Education*	High school or less	40	6.2	41.13	8.75
	Some college	84	12.9	40.36	7.80
	Bachelor's degree	409	62.9	35.30	7.30
	Advanced degree	117	18.0	34.11	6.69
Have a home pool*	No	333	51.2	39.18	7.88
	Yes	317	48.8	32.85	5.93
Know how to swim*	No	30	4.6	41.17	8.49
	Yes	620	95.4	35.85	7.56
**Have taken CPR**	No	190	29.2	35.76	7.82
	Yes	460	70.8	36.24	7.62
Relationship to toddler*	Parent/step/foster	520	80.0	36.70	7.78
	Grandparent	58	8.9	33.78	6.81
	Aunt/uncle/cousin	44	6.8	35.61	7.67
	Older sibling	28	4.3	30.46	2.87
Toddler gender*	Female	214	33.0	37.52	7.99
	Male	435	67.0	35.41	7.43
Toddler age*	1 yr	57	8.8	38.75	8.62
	2 yrs	159	24.5	36.06	7.48
	3 yrs	233	35.9	36.46	7.76
	4 yrs	201	30.9	34.96	7.28
Toddler had swim lessons*	No	265	40.8	38.43	8.03
	Yes	385	59.2	34.49	7.00
**How often swim with toddler**	Never	45	69.2	36.84	8.52
	A few times a summer	258	39.7	35.73	7.49
	A few times a month	177	27.2	35.58	7.48
	A few times a week	115	17.7	37.81	8.16
	Most days	55	8.5	35.27	7.10
**All Participants**		**650**	**100**	**36.10**	**7.68**

Note: Mean=mean supervision behavior score, SD=standard deviation, *=p<0.01 on one-way ANOVA

## Results

Results were analyzed for 650 participants. Most of the participants in the survey were parents/foster parents/step-parents of a toddler (80%). The majority were 25 - 34 yrs old (54%) and male (62%). Most participants reported their race and/or ethnicity to be white (71%). The majority had a household income of $50,000-$99,999 (51%) and a bachelor's degree (63%). There was at least one participant from every state in the U.S. except Alaska and South Dakota. See Table 1 for detailed demographic information. 

**Supervision behavior score**

The average supervision behavior score was 36.10 (SD=7.68), with a range of scores of 23 - 50 within the possible range of 10 - 50. The average supervision behavior score indicates attitudes between neutral (30) and agreement with attentive supervision behavior (40). Responses to individual statements used to calculate the total supervision behavior score are in Table 2. Responses to the water safety knowledge questions can be found in Table 3 and to the perceptions of arm’s reach supervision statements can be found in Table 4 .

**Table 2:** Percentage of responses to swimming supervision behavior statements.

Statements of distractible supervision	Strongly disagree	Disagree	Neutral	Agree	Strongly agree
I can still keep an eye on my toddler if they are swimming while I hang out with friends or family outside of the pool.	13%	14%	16%	43%	13%
If I am expecting an important text, I can leave my toddler in the pool for a minute to check my phone outside of the pool.	27%	18%	17%	26%	12%
If I'm grilling or need to prepare food near the pool, I can look after my toddler in the pool at the same time.	21%	19%	17%	29%	14%
When my toddler is swimming, I can read a book or scroll through social media on the side of the pool.	27%	16%	18%	26%	13%
If I need to take a bathroom break, I can run inside while my toddler is in the pool.	31%	14%	16%	28%	11%
**Statements of attentive supervision**	**Strongly disagree**	**Disagree**	**Neutral**	**Agree**	**Strongly agree**
I stay close to my toddler when they are in the pool and don’t step away even if there are things I really need to do nearby.	1%	4%	16%	39%	40%
I don't let anyone distract me from keeping an eye on my toddler when they are in the pool.	1%	6%	16%	43%	34%
If my toddler is in the pool, I would not leave them unattended even if I had an important phone call.	1%	6%	16%	36%	42%
I give my full attention to my toddler whenever they are in the pool.	0%	3%	12%	37%	47%
I don't leave my toddler alone in the pool even if it's just for a few minutes.	2%	6%	15%	34%	43%

**Table 3:** Percentage of correct responses to questions in knowledge section.

False statements	Correct
Water wings (i.e., inflatable arm floaties) are classified as life-saving flotation devices.	36%
Toddlers should only wear life jackets if they are in open water, such as lakes or oceans.	39%
If a child is drowning, you usually hear them splashing or calling for help.	49%
**True statements**	Correct
In the U.S., drowning is the leading cause of death due to injury for toddlers.	49%
A child can drown in less than a minute.	72%
In the U.S., the most common place for a toddler to drown is in a home swimming pool.	72%
Children can drown in swimming pools even when it is not swim time.	72%
Isolation fences around pools can prevent toddlers from drowning.	78%
If CPR is started immediately after removing an unconscious child from the water, it could make the difference between the child living and dying.	80%
When in a pool, it is recommended that toddlers are kept within arm’s reach at all times	83%

**Table 4:** Mean agreement with perceptions of arm’s reach supervision statements.

Statements	Mean agreement
Parents or caregivers should keep toddlers within arm's reach when they are in the pool.	4.07
Toddlers should be kept within arm's reach of their parent or caregiver even if a lifeguard is on duty.	4.00
Toddlers should be kept within arm's reach of their parent or caregiver even if they are playing with other toddlers.	3.99
Toddlers should be kept within arm's reach of their parent or caregiver even if they are wearing water wings (i.e., arm floaties).	3.97
Toddlers should be kept within arm's reach of their parent or caregiver even if they have had swimming lessons.	3.96
Toddlers should be kept within arm's reach of their parent or caregiver even if they are in the pool at a pool party.	3.95
Toddlers should be kept within arm's reach of their parent or caregiver even if they are wearing a coast guard approved flotation device (e.g., lifejacket or puddle jumper).	3.93
Toddlers should be kept within arm's reach of their parent or caregiver even if they are in water that is not above the toddler's head.	3.91
Toddlers should be kept within arm's reach of a parent or caregiver even if an older child (9-13 years old) is with them.	3.87
Toddlers should be kept within arm's reach of a parent or caregiver even if an older child (14-17 years old) is with them.	3.67

Note: 1=Strongly disagree, 5=Strongly agree

**Predictors of supervision behavior**

Fourteen variables included in the univariate analyses (Table 1) showed a significant association with supervision behavior and were included in the multivariate regression model. The subsequent multivariate linear regression model explained 57% of the variance in supervision behavior (F (29, 619) = 27.94, p<0.001, R2=0.5669). Five variables continued to show a significant association with supervision behavior after multivariate adjustment (Table 5 ). High water safety and drowning knowledge was predictive of attentive supervision behavior. Additionally, positive perceptions of arm’s reach supervision was predictive of attentive supervision behavior. Attentive supervision behavior was also predicted by having a toddler with fewer water competency skills. Having an advanced degree was predictive of less attentive supervision behavior when compared to having a high school education or less. Having a home pool was also predictive of less attentive supervision behavior.

**Table 5:** Linear regression model results for swimming supervision behavior score.

Variable	Category	Coeff.	Std. Err.	p-value	[95% CI]		𝛽
**Knowledge**		1.28	0.12	<0.01	1.05	1.51	0.36
**Perceptions of arm's reach supervision**		0.30	0.03	<0.01	0.23	0.36	0.27
**Gender**	Female						
	Male	-0.34	0.47	0.47	-1.27	0.59	-0.02
**Age**	18 - 24 yrs						
	25 - 34 yrs	0.49	1.14	0.67	-1.75	2.73	0.03
	35 - 44 yrs	1.53	1.17	0.19	-0.78	3.83	0.09
	45 - 54 yrs	1.27	1.37	0.35	-1.42	3.95	0.04
	55 yrs or older	0.08	1.54	0.96	-2.94	3.10	0.00
**Race/ethnicity**	White						
	Asian	0.25	1.29	0.84	-2.28	2.78	0.01
	Black	-0.32	0.56	0.57	-1.42	0.78	-0.02
	Hispanic/latino	1.23	0.98	0.21	-0.70	3.15	0.03
	Mixed race	0.73	1.17	0.53	-1.56	3.02	0.02
**Household income**	Less than $25,000						
	$25,000-$49,999	-0.10	0.89	0.91	-1.85	1.66	-0.01
	$50,000-$99,000	-0.13	0.87	0.88	-1.85	1.59	-0.01
	$100,000 or more	1.93	1.03	0.06	-0.09	3.95	0.08
**Education**	High school or less						
	Some college	-1.64	1.02	0.11	-3.64	0.37	-0.07
	Bachelor's degree	-2.25	0.91	0.01	-4.04	-0.45	-0.14
	Advanced degree	-3.41	1.01	<0.01	-5.39	-1.42	-0.17
**Have a home pool**	No						
	Yes	-2.01	0.47	<0.01	-2.94	-1.09	-0.13
**Know how to swim**	No						
	Yes	-0.84	1.03	0.42	-2.86	1.18	-0.02
**Relationship to toddler**	Parent/step/foster						
	Grandparent	-0.73	0.82	0.37	-2.33	0.87	-0.03
	Aunt/uncle/cousin	-0.39	0.82	0.64	-2.00	1.22	-0.01
	Older sibling	-0.53	1.08	0.62	-2.64	1.58	-0.01
**Toddler gender**	Female						
	Male	-0.35	0.47	0.46	-1.28	0.58	-0.02
**Toddler age**	1 yr						
	2 yrs	0.84	0.83	0.31	-0.78	2.47	0.05
	3 yrs	1.12	0.79	0.16	-0.45	2.68	0.07
	4 yrs	0.75	0.83	0.37	-0.88	2.38	0.05
**Toddler had swim lessons**	No						
	Yes	0.20	0.50	0.69	-0.78	1.17	0.01
**Toddler water competency**		-0.88	0.13	<0.01	-1.14	-0.62	-0.22

Note: Coeff.= coefficient, Std. Err.=standard error, CI=confidence interval

## Discussion

The results of this study show that, in general, parents or caregivers of toddlers may not be using supervision approaches that offer the best protection from drowning. Many caregivers showed a willingness to engage in activities like talking on the phone or grilling while supervising their toddler at a pool, with some willing to leave the pool area to use the restroom. Additionally, results showed that attentive caregiver supervision was predicted by high water safety knowledge and positive perceptions of arm’s reach supervision, while less attentive supervision was predicted by having an advanced degree, having a home pool, and reporting a high number of water competency skills for their toddler.

Despite most caregivers reporting staying close to their toddler and giving them full attention when they are swimming when asked to agree with statements worded positively towards attention, when confronted with statements worded positively towards distractible behaviors, caregivers were more split about their agreement (Table 2). The seemingly contradictory responses between the distractible and attentive supervision behavior statements suggests that caregivers believe they are being attentive even when they allow themselves to step away to attend to other things while they are supervising their toddler in a pool. This shows that common attitudes about what type of supervision is necessary for toddlers when swimming may underestimate the need for close, attentive, and continuous supervision, as advised by the AAP. Because drowning can happen in under a minute, leaving a toddler with absent or poor quality supervision for any length of time can be fatal.^5^

Previous research on supervision around bodies of water, generally, suggests that parents may not provide adequate supervision because they do not believe that it is possible or necessary.^10^ Despite the higher risk of drowning fatalities than other unintentional injury categories for toddlers,^1^ research shows that mothers believe their toddler can manage the risk of drowning themselves more than they can manage the risks of other injuries, such as burns, falls, and poisoning.^16^ The common belief that children can manage their own risk is supported by our finding that toddlers believed to have more water competency skills might be offered less attentive supervision than toddlers believed to have few water competency skills. Modulating supervision based on beliefs about water competency or swimming ability is a hazardous practice since no child is drown-proof. Adding to the risks posed by this misconception, parents may overestimate their child’s swimming ability.^17^

Our findings did not support previous research showing that parents may believe that their child needs less supervision following swim lessons.^10^ Additionally, unlike prior research, child age was not found to be a predictor of supervision behavior.^8,13^ Although lessening supervision for toddlers is never advised, it makes sense that supervision is more likely to be moderated by the toddler’s water competency rather than by whether they have had swimming lessons or their age since lessons and age are not guarantees of a high water competency.

Regression 𝛽 results indicate that the two factors in the model that are most predictive of attentive supervision behavior are having greater drowning and water safety knowledge and having positive perceptions of arm’s reach supervision. These findings support the need for education campaigns for parents and caregivers that increase knowledge of drowning and what constitutes quality supervision for children when they are swimming. Research shows that parent education programs running concurrently with children’s swimming lessons can counter the misconception that toddlers need less supervision if they have more water competency skills, can increase water safety knowledge, and can change perceptions about supervision needs and drowning risk.^18,19^ The S.A.F.E.R. Near Water program, developed in Canada, showed that parent seminars held during swimming lessons, combined with posters at the pool were effective at increasing knowledge and changing perceptions.^19^ Research by Glassman et al. on a social marketing intervention used in the U.S. with parents of inner-city youth during swim lessons was shown to be effective using brochures, emails, text messages, postcards, and facebook posts.^18^

Additionally, drowning prevention messaging should address supervision in addition to the other layers of protection recommended by the AAP, such as improving home pool barriers, taking swim lessons, getting CPR training, and using personal flotation devices.^5^

Increasing knowledge and changing perceptions about drowning risk and supervision has been shown to change supervision behavior. An intervention at public pools in Australia that used pool signage, information cards, lifeguard training about communicating the importance of supervision to patrons, and available website information improved observed supervision behavior.^20^ Together, research on drowning prevention interventions suggests that a range of approaches may be effective and that it may be important to base intervention approaches in behavioral theory, such as guided by the Health Belief Model.^18,19,20,21^ Additionally, drowning prevention messaging should address supervision in addition to the other layers of protection recommended by the AAP, such as improving home pool barriers, taking swim lessons, getting CPR training, and using personalflotation devices.^5^

Despite high water safety knowledge being an indicator of attentive supervision behavior, education level impacted supervision in an unpredicted way. Our results showed that supervision behavior was most attentive for those with the lowest education level and that averages progressively indicated less attentive supervision behavior as education level increased. It is unclear what might drive the impact of education on supervision in this direction. However, this finding is important to note since water safety education programming may target certain demographics under an assumption that the more educated a parent is, the more knowledgeable they might be about water safety and the more attentive they might be when supervising. Our results highlight how we should not make assumptions about what groups are most in need of drowning prevention education and messaging about water safety and the need for quality supervision.

It is well-known that home pools pose a risk to children. For toddlers, the majority of fatal toddler drownings occur in home pools.^4^ When trying to improve drowning prevention, it is generally understood that a lack of security devices and barriers, like pool fencing, contributes to home pool drowning incidence.^22^ However, our findings suggest that home pools may also pose a risk during planned swim times because caregivers with home pools may not be as attentive as caregivers without home pools. Possibly the constant pool accessibility and potentially high frequency of use can lull parents and caregivers into downplaying the risks. Our results suggest that pool owners are a key group to target with information about how to provide quality supervision when children are in or near a pool. 

The multi-variable regression model predicting caregiver supervision behavior at swimming pools accounted for over 50% of the variance in behavior. It is unclear what additional unmeasured factors might affect supervision. Because supervision behavior was self-report, there was no measure of environmental factors that might affect supervision, like the presence of lifeguards. Prior research observing supervision at public pools, where lifeguards were present, showed that supervision behavior was predicted by some situational factors, such as whether the child was playing with other children and by child age.^13^ Additionally, we did not assess psychosocial drivers of behavior. Studies of parents of toddlers show that psychosocial characteristics, such as anticipated regret, are predictive of parent supervision intentions and behaviors regarding home pools.^23,24^ Future research should further investigate what shapes pool supervision with observational studies and should include additional environmental and psychosocial factors. 
